# Expression of E-selectin ligands on circulating tumor cells: cross-regulation with cancer stem cell regulatory pathways?

**DOI:** 10.3389/fonc.2012.00103

**Published:** 2012-08-20

**Authors:** Monica M. Burdick, Karissa A. Henson, Luis F. Delgadillo, Young Eun Choi, Douglas J. Goetz, David F. J. Tees, Fabian Benencia

**Affiliations:** ^1^ Department of Chemical and Biomolecular Engineering, Russ College of Engineering and Technology, Ohio UniversityAthens, OH, USA; ^2^ Biomedical Engineering Program, Russ College of Engineering and Technology, Ohio UniversityAthens, OH, USA; ^3^ Department of Physics and Astronomy, College of Arts and Sciences, Ohio UniversityAthens, OH, USA; ^4^ Department of Biomedical Sciences, Heritage College of Osteopathic Medicine, Ohio UniversityAthens, OH, USA

**Keywords:** circulating tumor cells, cancer stem cells, epithelial-to-mesenchymal transition, selectins, selectin ligands, cell adhesion

## Abstract

Although significant progress has been made in the fight against cancer, successful treatment strategies have yet to be developed to combat those tumors that have metastasized to distant organs. Poor characterization of the molecular mechanisms of cancer spread is a major impediment to designing predictive diagnostics and effective clinical interventions against late stage disease. In hematogenous metastasis, it is widely suspected that circulating tumor cells (CTCs) express specific adhesion molecules that actively initiate contact with the vascular endothelium lining the vessel walls of the target organ. This “tethering” is mediated by ligands expressed by CTCs that bind to E-selectin expressed by endothelial cells. However, it is currently unknown whether expression of functional E-selectin ligands on CTCs is related to cancer stem cell regulatory or maintenance pathways, particularly epithelial-to-mesenchymal transition and the reverse, mesenchymal-to-epithelial transition. In this hypothesis and theory article, we explore the potential roles of these mechanisms on the dynamic regulation of selectin ligands mediating CTC trafficking during metastasis.

## INTRODUCTION

Distant metastasis is the culmination of an elaborate cascade of events in which cancer cells break away from the primary tumor, intravasate through blood vessel walls into the circulatory system, travel throughout the body, and finally extravasate through the vessels of a distant organ to establish a secondary colony. While resident in the blood vasculature, circulating tumor cells (CTCs) must survive biochemical and biophysical assaults inducing necrosis or apoptosis, plus avoid elimination by immune cells, in order to metastasize. Regardless of their ultimate fate, the clinical interpretation of CTCs arising from solid tumors has been the subject of much debate, with definitive answers yet to emerge as to if, when, and for which cancers these cells offer significant diagnostic, prognostic, or therapeutic value. Despite lack of consensus on their clinical utility, CTCs can still provide a meaningful portrait of a cancer patient’s health, or rather disease, status. CellSearch, a test marketed by Johnson & Johnson’s Veridex division, is FDA-approved to capture and enumerate CTCs in metastatic breast, colon, and prostate cancer patients for prognostic purposes (Dawood et al., 2008; Mostert et al., 2009; Riethdorf and Pantel, 2010). More recently, the development of next-generation fluidics-based CTC isolation devices by the Haber and Toner groups, the CTC-chip and herringbone (HB)-chip (Nagrath et al., 2007; Stott et al., 2010; Yu et al., 2011), has generated increased attention to CTCs and the use of “liquid biopsies” or “blood biopsies” to enumerate and capture CTCs for further study. As with any portrait, further examination reveals nuances not observed at first glance. For instance, post-capture investigation using RT-PCR in the AdnaTest (AdnaGen) may reveal upregulated pathways related to cancer stem cells (CSCs), metastatic aggressiveness, or responsiveness to treatment (i.e., trastuzumab for HER-2 overexpressing breast cancers) that are impossible to observe through a simple CTC count (Fehm et al., 2007; Dawood et al., 2008; Riethdorf and Pantel, 2008, 2010; Mostert et al., 2009). Though the scientific and medical communities may achieve significant new insights from these blood biopsies, the information itself is static. Cancer is dynamic. How medical professionals interpret a particular patient’s case, as well as predict future outcomes of an ever-changing disease, will depend partially on information gleaned from CTC assessments at single moments in time.

In general, CTCs possessing enhanced survival capabilities will generate metastatic colonies in distant organs, as well as reseed the original primary tumor with more aggressive cells (Kim et al., 2009). Uncovering the molecular mediators by which CTCs initiate adhesion with endothelial cells lining the blood vessel walls of the target site may therefore prove useful in predicting and thwarting metastasis. In particular, stimulated vascular endothelium expressing E-selectin can capture CTCs expressing E-selectin ligands, thereby initiating adhesion and subsequent CTC invasion. However, this statement is a simplification of a tangle of issues underlying functional selectin ligand expression on cancer cells. To be qualified as a true selectin ligand, Varki (1997) proposed that the purported ligand must be expressed “in the right place at the right time” among other criteria. So do all CTCs express selectin ligands, or even the “right” selectin ligands? How and when do these selectin ligands arise? Are they modulated by pathways associated with epithelial-to-mesenchymal transition (EMT) or other mechanisms of CSC generation and maintenance, or are they independent of these pathways? In this article, we explore the complex networks through which selectin ligands on CTCs may be regulated and propose working theories based on ongoing studies with breast cancer in our laboratories. New findings from these investigations, coupled with additional discoveries from other labs, will address significant shortcomings in our understanding of the molecular networks promoting cancer metastasis.

## CTCs AND CELL ADHESION MEDIATED BY E-SELECTIN AND ITS LIGANDS

**FIGURE 1:**
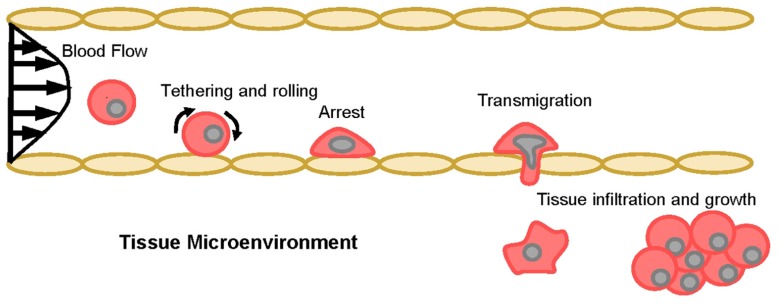
**The multi-step model of cell adhesion and migration in blood vessels.** Schematic representation of the cascade of events involved in cellular transit from the vasculature into the underlying tissue space. Tethering recruits circulating cells from the bulk flow stream, then rolling slows lateral translocation across the endothelium and facilitates firm arrest. If the correct cytokine and/or chemokine gradients are present, adherent cells will transmigrate through the endothelium and infiltrate the tissue microenvironment.

It has been proposed that the early steps by which CTCs cells leave the bloodstream to invade secondary sites mimic the physiologic trafficking of leukocytes to sites of inflammation and hematopoietic stem cells to bone marrow. Because numerous excellent review articles on cell trafficking have been published through the years (Springer, 1994; Sackstein, 2005; Barthel et al., 2007; Konstantopoulos and Thomas, 2009; Zarbock et al., 2011; Bendas and Borsig, 2012; Chase et al., 2012; Geng et al., 2012), only a general overview is presented here (**Figure 1**). Circulating cells are first captured or “tethered” from bulk blood flow onto vascular endothelial cells, which is immediately followed by rolling on the endothelium. Tethering and rolling are typically mediated by interactions between ligands expressed on the surface of the circulating cells that recognize E-selectin, an endothelial adhesion molecule upregulated in response to inflammatory stimuli as well as constitutively expressed by bone and dermal endothelial cells (Springer, 1994; Sackstein, 2004). Subsequently, rolling cells firmly adhere and migrate through the vessel wall into the underlying tissue in response to specific cytokines and chemokines.

Therefore, this multi-step model indicates that CTCs must initially tether on endothelial cells, presumably through E-selectin ligand recognition of E-selectin, in order to trigger the series of events necessary for metastatic growth. These adhesive interactions occur under hydrodynamic shear stresses generated by blood flow (post-capillary venule and bone marrow endothelial venule wall shear stress ranges from 0.5 to 4.0 dyn/cm^2^; Jones et al., 1991; Mazo et al., 1998), enabled by the hallmark catch-slip bonds and rapid bond formation/breakage kinetics of selectins and their ligands (Dembo et al., 1988; Marshall et al., 2003; Zhu and McEver, 2005; Evans and Calderwood, 2007; Ham et al., 2007; McEver and Zhu, 2007). E-selectin has been established as a mediator of colon and prostate cancer adhesion and distant metastasis (Khatib et al., 2002; Barthel et al., 2007, 2009), and there is clinical and *in vitro* evidence for the role of E-selectin in promoting metastasis of several other cancers, including breast, pancreatic, and head and neck cancers (Wenzel et al., 1995; Eshel et al., 2000; Barthel et al., 2007; Geng et al., 2012). The other two members of the selectin family, P-selectin expressed by activated platelets and activated endothelium and L-selectin expressed by most leukocytes, also have been proposed to participate in cancer metastasis (Laubli and Borsig, 2010; St Hill, 2012).

Notably, the expression levels of the minimal selectin-binding epitopes sialyl Lewis X (sLe^X^,NeuAcα(2,3)Galβ(1,4)[Fucα(1,3)]GlcNAc) and its stereoisomer sialyl Lewis A (sLe^A^, NeuAcα(2,3)Galβ(1,3)[Fucα(1,4)]GlcNAc) on certain glycoproteins and glycolipids increase progressively from normal tissue to early stage cancer to metastatic disease, consistent with aberrant glycosylation rendering altered cell adhesion molecules relative to normal tissue in most cancers, including breast, bladder, and colon cancers (Izumi et al., 1995; Klopocki et al., 1996; Renkonen et al., 1997; Skorstengaard et al., 1999; Kajiwara et al., 2005). Transfer of sialic acid (NeuAc) onto a terminal galactose (Gal) residue occurs through the action of α(2,3) sialyltransferases. The enzymes directing α(1,3) fucosylation for sLe^X^ production are multiple-fucosyltransferases (FTs) III, IV, V, VI, and VII while FTIII and FTV are also α(1,4) FTs involved in the production of sLe^A^ (Edbrooke et al., 1997; de Vries et al., 2001; Dupuy et al., 2004). Clearly, these enzymes must be (dys)regulated in cancer cells through the transition from primary tumor to advanced stage cancer to result in the observed upregulation of sLe^X/A^ and thus selectin ligands (Renkonen et al., 1997; Matsuura et al., 1998). Although the tumor stroma and hypoxic conditions are known to influence tumor cell glycosylation (Stern et al., 2001, 2002; Kannagi, 2004), the exact biochemical (or biophysical) regulators of cancer glycosylation are unknown. Nevertheless, the presence of sialofucosylated moieties such as sLe^X/A^ is significant in that upregulated expression of functional selectin ligands may indicate their role in promoting CTC adhesion during metastasis (Burdick et al., 2001; Kannagi et al., 2004; Barthel et al., 2007). Thus, it is necessary to identify the core proteins or lipids presenting sialofucosylated glycans to better characterize roles for specific selectin ligands.

To date, several major tumor cell surface glycoprotein selectin ligands that may fulfill the criteria of “real” selectin ligands have been identified, most prominently the specialized CD44 glycoform HCELL as an E-/L-/P-selectin ligand on colon cancer cells (Hanley et al., 2005, 2006; Burdick et al., 2006), and an E-selectin ligand on prostate and breast cancer cells (Barthel et al., 2009; manuscript in preparation). Carcinoembryonic antigen (CEA, CD66) and podocalyxin-type protein-1 (PCLP-1) have also been named E-selectin ligands expressed on colon and prostate cancer cells (Barthel et al., 2009; Thomas et al., 2009). On breast cancer cells, CD24 acts as a P-selectin ligand but not an E-selectin ligand (Aigner et al., 1998), and Mac-2bp acts as an E-selectin ligand (Shirure et al., 2012). Additional mucinous proteins, such as MUC-1, CD43, and PSGL-1, have also been proposed as selectin ligands on a variety of cancer cells (Barthel et al., 2007; Geng et al., 2012). Contributory roles have also been identified for colon, prostate, breast, and head and neck cancer sialofucosylated glycolipids in adhesion to endothelial E-selectin (Burdick et al., 2003; Dimitroff et al., 2004; Barthel et al., 2007; Shirure et al., 2011; Geng et al., 2012). Though the understanding of selectins and their ligands is growing, it is imperative to consider their functionalities in the wider context of biochemical and biophysical factors encountered by CTCs in transit.

## CTC TRANSIT THROUGH CAPILLARIES

The ability of cancer cells to enter small vessels such as capillaries (as well as to roll in larger vessels such as post-capillary venules described above) depends critically on the mechanical deformability of the cells. Capillaries range from 2 to 8 μm in diameter (Doerschuk et al., 1993) and cancer cells, which tend to be large and stiff, may not be able to deform enough to enter at least some portions of the capillary bed (Liotta, 1987; Weiss et al., 1988; Chambers et al., 1992; Lafrenie et al., 1993). Organs with small vessels that are susceptible to metastasis include the lung microcirculation (which is particularly important because it is the first capillary bed that a metastasizing cancer cell entering the venous circulation will encounter after passing through the first two chambers of the heart), bone marrow and liver sinusoids, and the kidney microcirculation. The mechanical properties of cancer cells surely play a role in transit: if certain CTCs are stiff and resistant to deformation, then the possibility of sequestration at the entrance of small vessels should be large. Conversely, if CTCs are less stiff (more deformable), then their potential to pass through the microcirculation and metastasize could be enhanced. Furthermore, it is possible that deformation is not a by-stander process for the cell; deformation itself may induce changes affecting molecular and mechanical phenotype, perhaps in a manner that promotes CTC survival and metastasis.

**FIGURE 2:**
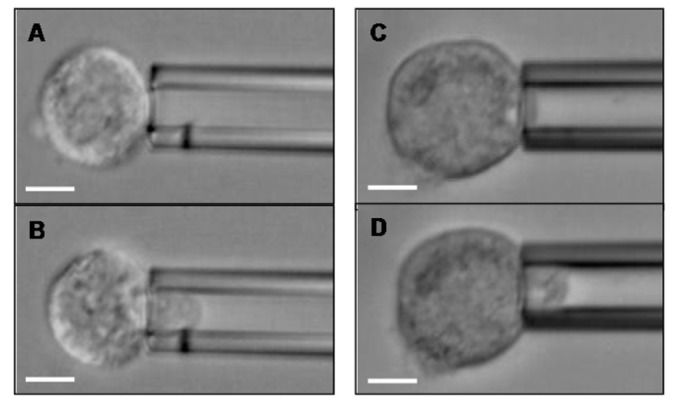
**Breast cancer cells can be aspirated into a glass micropipette.** A CD44^+^/CD24**^-^ Hs578T breast cancer cell **(A)** before and **(B)** after partial aspiration into a micropipette of 8.4 μm diameter. A CD44^+^/CD24^+^ BT-20 breast cancer cell **(C)** before and **(D)** after partial aspiration into a 9.0 μm diameter micropipette. Scale bar is 10 μm in all Figures.

Protocols to quantify cellular mechanical properties have existed for nearly 30 years, and parameters for models of cell mechanics have been measured using many experimental techniques: micropipette aspiration (**Figure 2**), magnetic twisting rheometry, cell stretching with optical tweezers or mechanical stretching devices, nanoscale indentation with probes or AFM tips, particle tracking microrheology, etc. (Mason and Weitz, 1995; Shroff et al., 1995; Choquet et al., 1997; Mason et al., 1997; Thoumine and Ott, 1997; Bausch et al., 1999; Yap and Kamm, 2005; Sirghi et al., 2006). As a result of these efforts, much is known about the deformability of red and white blood cells (which are known to undergo massive deformations in the normal course of circulation) and a sampling of other cell types. On the basis of these collective works (Mason and Weitz, 1995; Shroff et al., 1995; Choquet et al., 1997; Mason et al., 1997; Thoumine and Ott, 1997; Bausch et al., 1999; Yap and Kamm, 2005; Sirghi et al., 2006), it was found that the major distinction in cell rheological properties is whether the cell behaves like a liquid drop with a cortical tension (as white blood cells clearly do) or as a viscoelastic solid (most other cell types). Devices to identify cell subsets based on differences in cellular mechanical properties are in early development stages (Oakey et al., 2010; Sraj et al., 2010), and these methodologies are being considered for identifying and isolating normal healthy mesenchymal stem cells (MSCs) for use as therapeutics and in regenerative medicine (Porada et al., 2006; Parekkadan and Milwid, 2010). These cells lack unique cell surface molecules through which they can be easily isolated from their sources (e.g., bone marrow, umbilical cord; Porada et al., 2006; Pountos et al., 2007) but have distinct mechanical properties compared to their differentiated daughter cells. These differences are currently being explored as specific identifying MSC characteristics (Darling et al., 2008; Tan et al., 2008; Yu et al., 2010). Similarly, benign versus tumorigenic cancer cells have been explored for differing traits (Kim et al., 2008; Hou et al., 2010). However, much more work needs to be performed to understand CTC metastatic potential attributable to inherent or alterable molecular and mechanical properties. It is tantalizing to speculate a role for biophysical modulation of CTC properties, including effects on selectin ligand expression or function.

## CSCs, EMT, AND MESENCHYMAL-TO-EPITHELIAL TRANSITION

The discovery and identification of leukemic stem cells (LSCs) effectively ushered in a new era of cancer research (Lapidot et al., 1994; Bonnet and Dick, 1997). LSCs share the properties of self-renewal and pluripotency with their normal hematopoietic stem cell brethren, but are also leukemogenic. LSCs are particularly dangerous in that they can survive chemotherapy (Costello et al., 2000; Graham et al., 2002; Holtz et al., 2002), leading to relapse with LSCs even more aggressive than their previous incarnation (Oravecz-Wilson et al., 2009). Shortly after LSC identification, a groundbreaking report by Al-Hajj et al. (2003) found that breast cancers similarly harbor deadly CSCs, which exhibited a much greater propensity for tumor formation than cells of a different phenotype. These breast CSCs were putatively characterized by the expression levels of glycoprotein markers on the surface of the cell: high expression of CD44, little to no expression of CD24, high expression of epithelial-specific antigen (ESA), and lack of lineage markers (lin), or CD44^+^/CD24^-/low^/ESA^+^/lin^-^ (Al-Hajj et al., 2003). These CSCs were able to form heterogeneous tumors from a relatively small number of cells. Specifically, only 200 CD44^+^/CD24^-/low^/ESA^+^/lin^-^ breast cancer cells, isolated from patient primary tumors, could regenerate and expand to form secondary tumors that also contained CSCs, in as little as 12 weeks in mice (Al-Hajj et al., 2003). In contrast, as many as 20,000 cells of alternate phenotypes from the same tumor origin as the CD44^+^/CD24^-/low^/ESA^+^/lin^-^ cells were unable to form new tumors. Thus, the breast CSCs were capable of self-renewal and differentiation, two general properties possessed by normal stem cells, and the ability to generate new tumors (Al-Hajj et al., 2003; Ponti et al., 2005; Fillmore and Kuperwasser, 2008). Since this initial breast cancer study, CSCs have reportedly been found in nearly all solid cancers, with a specific molecular phenotype for each type of cancer. However, the cancer research community continues to debate the true nature of CSCs (Campbell and Polyak, 2007; Gupta et al., 2009; Badve and Nakshatri, 2012; Liu et al., 2012; Magee et al., 2012), including whether CSCs are tumor-initiating or metastasis-initiating cells (Kelly et al., 2007; Adams and Strasser, 2008; Fillmore and Kuperwasser, 2008). The reasons for the extended scientific discussion are many and are outlined in a comprehensive review from the Morrison lab (Magee et al., 2012).

Perhaps some of the confusion and seemingly contradictory findings surrounding CSCs will be allayed by the growing evidence demonstrating that CSCs are not a single population of cells identified by one specific molecular signature. Rather, while all CSCs possess general stem cell properties, CSCs are actually comprised of heterogeneous subpopulations with multiple molecular and functional phenotypes that are generated through different pathways (Liu et al., 2012; Magee et al., 2012). It is becoming abundantly clear for breast cancer that such heterogeneity exists in its CSCs. Breast CSCs that are CD44^+^/CD24^-^ (the simplified breast CSC phenotype) are the result of cytokine-induced EMT (Mani et al., 2008; Morel et al., 2008; Blick et al., 2010; Liu et al., 2012), a process by which cells lose epithelial characteristics (E-cadherin expression, cell–cell contacts, polarity) and become more mesenchymal (N-cadherin expression, mesenchymal morphology, enhanced migration abilities; Onder et al., 2008; Zeisberg and Neilson, 2009). Many of the properties EMT confers are normally helpful to development (Kalluri and Weinberg, 2009), but EMT can also contribute to cancer progression in adult tissue (Mani et al., 2008; Onder et al., 2008). Often, cancer cells at the invasive front of a primary tumor have a mesenchymal phenotype (Kalluri and Weinberg, 2009). Interestingly, in breast cancer patients with metastases, CTCs have been found to express markers of EMT in addition to stem cell traits (Aktas et al., 2009; Bonnomet et al., 2010; Kallergi et al., 2011). It is important to note that EMT is reversible, such that cells can undergo mesenchymal-to-epithelial transition (MET). The Wicha group reported that CSCs can exist in an MET state (Liu et al., 2012) as well as an EMT state previously found by the Weinberg group (Mani et al., 2008; Liu et al., 2012). MET CSCs actively self-renew and express aldehyde dehydrogenase (ALDH, a marker independently identified as a CSC indicator in several types of cancer (Ginestier et al., 2007; Clay et al., 2010; Silva et al., 2011; Kryczek et al., 2012), epithelial cell adhesion molecule (EpCAM, the same molecule that forms the basis for the capture of CTCs by CellSearch and the CTC- and HB-chips), and CD49f (α_6_ integrin subunit) in contrast to quiescent yet invasive CD44^+^/CD24^-^/EpCAM^-^/CD49f^+^ EMT CSCs (Liu et al., 2012). Given the interconversions between CSC states, which are regulated by microRNAs (miRNAs), it is not surprising that there exists a subpopulation of CD44^+^/CD24^-^ and ALDH^+^cells (Liu et al., 2012). However, studies linking CSCs with properties facilitating CTC lodgment at sites of metastasis (i.e., selectin ligands and cell mechanical properties) are lacking.

## PUTTING IT ALL TOGETHER: HYPOTHESIZED BREAST CANCER MODELS LINKING REGULATION OF CSCs, CTCs, AND E-SELECTIN LIGANDS

Arguably, CSCs and CTCs from breast cancer are the most well-studied among all cancers, thereby easing efforts aimed at uncovering crosstalk between CSC regulatory pathways, CTC characteristics, and expression of functional selectin ligands. Such investigations may aid in diagnosing breast cancer at an early stage, when it is largely considered curable, or assist in identifying new therapeutic targets or treatment modalities for those women diagnosed at the metastatic stage, for whom the 5-year survival rate is ~20% (DeSantis et al., 2011). Most commonly, breast cancer metastases are found in the lungs and bone marrow (Moore, 2001; Minn et al., 2005; Balic et al., 2006; Riethdorf and Pantel, 2010), exhibiting a tropism not explainable by circulation pattern alone (Minn et al., 2005; Talmadge and Fidler, 2010). Recently, it has been reported that disseminated breast cancer cells in human bone marrow are largely CD44^+^/CD24^-^ (Abraham et al., 2005; Balic et al., 2006), corresponding to EMT CSCs. These CD44^+^/CD24^-^ are also resistant to radiotherapy and chemotherapy (Diehn and Clarke, 2006; Phillips et al., 2006; Reim et al., 2009). It is therefore necessary to understand the reasons for CD44^+^/CD24^-^ breast cancer cells in bone: whether CTCs are CD44^+^/CD24^-^ CSCs that preferentially migrate and establish metastases, or if non-CD44^+^/CD24^-^ CTCs are induced to the CD44^+^/CD24^-^ phenotype in the bone marrow.

**FIGURE 3:**
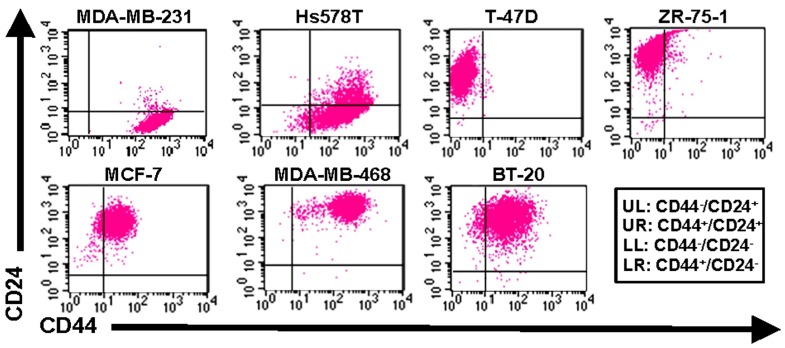
**Expression of CD44 and CD24 on breast cancer cell lines.** Flow cytometric analysis of cell surface expression on human breast cancer cells from the American Type Culture Collection (ATCC). Cells were simultaneously stained with CD44-FITC and CD24-PE mAbs and analyzed on a BD FACSort cytometer/sorter. Quadrants were set using appropriate FITC or PE-labeled isotype controls. The upper left quadrant represents CD44^-^/CD24^+^ cells, upper right quadrant represents CD24^+^/CD24^+^, the lower right quadrant represents CD44^+^/CD24**^-^ cells (i.e., phenotype of purported breast cancer stem cells), and lower left quadrant represents CD44^-^/CD24^-^. Data are representative of *n* = 6 independent experiments.

**FIGURE 4:**
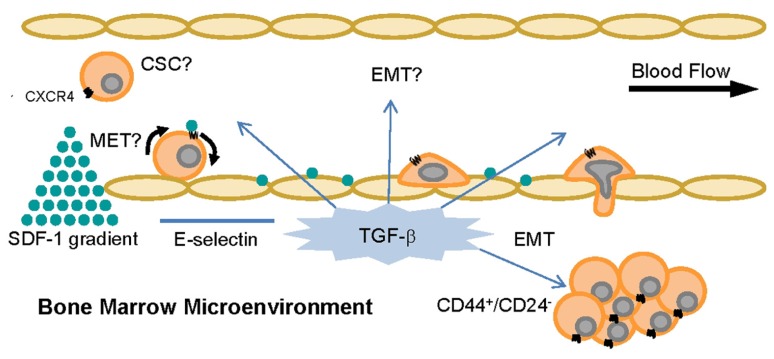
**TGF-β and SDF-1 can alter CTC phenotype and behavior in the bone.** The cytokine TGF-β and the chemokine SDF-1 are constitutively expressed in the bone microenvironment and control pathways related to cell adhesion and homing, EMT, MET, cell cycle, and tumorigenicity. It is currently unknown if inducible EMT or MET affects cancer cell expression of functional E-selectin ligands and thus CTC capacity to metastasize.

As mentioned previously, E-selectin is constitutively expressed on bone marrow endothelium (Keelan et al., 1994; Schweitzer et al., 1996), and breast cancer cells have been shown to express E-selectin ligands on their surface (Tozeren et al., 1995; Narita et al., 1996; Zen et al., 2008; Julien et al., 2011; Shirure et al., 2011, 2012). Previous studies have also demonstrated the E-selectin-dependence of binding interactions between commercially available breast cancer cell lines and human umbilical vein endothelial cells (HUVECs; Giavazzi et al., 1993; Narita et al., 1996; Julien et al., 2011; Shirure et al., 2011, 2012). As the expression levels of the minimal selectin-binding epitopes sLe^X^ and sLe^A^ increase progressively from normal tissue to early stage breast cancer to metastatic disease (Renkonen et al., 1997), it may be hypothesized that CTCs retain expression of selectin ligands that were generated in the primary site, then upregulate such ligands during transit to the metastatic site. Altogether, these findings imply that E-selectin and its ligands are likely to comprise important elements of breast cancer metastasis *in vivo*. Since breast cancer cells at the invasive front of a primary tumor tend to be mesenchymal (Kalluri and Weinberg, 2009) and breast CTCs have been found to express markers of EMT in addition to stem cell traits (Aktas et al., 2009; Bonnomet et al., 2010), it would seem a logical extension of the hypothesis that E-selectin ligands are upregulated with EMT and the corresponding CD44^+^/CD24^-^ CSC phenotype. However, our studies with human breast cancer cell lines revealed surprising results: non-CD44^+^/CD24^-^ cells expressed much greater E-selectin ligand activity than CD44^+^/CD24^-^ cells (**Figure 3** and **Table 1**; Shirure et al., 2011, 2012; manuscript in preparation). These findings imply that lower expression of E-selectin ligands correlates with CD44^+^/CD24^-^ breast CSCs arising from EMT. Notably, the bone marrow microenvironment is enriched in TGF-β, a cytokine that is well-known to induce EMT (Brown et al., 2004; Lee et al., 2008; Mani et al., 2008; Lenferink et al., 2010), and production of TGF-β by microenvironment stromal cells may be responsible for CD44^+^/CD24^-^ breast cancer cells in bone (Abraham et al., 2005; Balic et al., 2006). Thus, it may be speculated that soluble TGF-β decreases the expression of E-selectin ligands either before or during CTC engagement with bone marrow endothelium (**Figure 4**), thus throwing into doubt the relevance of E-selectin ligands on breast CTCs in establishing bone metastases. Studies in which EMT is induced in breast cancer cells need to be performed, with coordinated monitoring of glycosylation machinery, core E-selectin ligand protein and lipid expression, E-selectin ligand activity under flow conditions, and EMT and CSC markers, in order to verify or refute mechanistic links between functional E-selectin ligand expression and transition/maintenance of CD44^+^/CD24^-^ CSCs. Ultimately, it may be found that downregulation of E-selectin ligands is not dependent on EMT *per se*, since E-selectin ligand activity fails to decrease consistently from the least mesenchymal luminal to the somewhat mesenchymal basal A to the most mesenchymal basal B cells (**Table 1**). Instead, persistent suppression of E-selectin ligands in CD44^+^/CD24^-^ CSCs may be controlled by EMT pathways.

**Table 1:** Expression of CD44, CD24, and E-selectin ligands on human breast cancer cell lines.

Cell line	CD44/CD24 status	Subtype	E-selectin ligand activity
MDA-MB-231	+/low	Basal B	+
Hs578T	+/low	Basal B	+
ZR-75-1	low/+	Luminal	+++
T-47D	low/+	Luminal	++
MCF-7	+/+	Luminal	+++
MDA-MB-468	+/+	Basal A	+++
BT-20	+/+	Basal A	++++

*Column 1: Human breast cancer cell lines from ATCC. Column 2: Flow cytometric analysis of cell surface expression on breast cancer cells. Key: – is <2% positive (i.e., same intensity as isotype control antibody), low is 2–10% positive, moderate is 10–80% positive, + is >80% positive. Column 3: Breast cancer cell subtype as reported by Neve etal. (2006). Column 4: E-selectin ligand activity was assessed in the parallel plate flow chamber (Shirure etal., 2011, 2012; and manuscript in preparation). Key: +, upper limit of E-selectin-dependent tethering (i.e., recruitment from fluid flow) to IL-1β-stimulated HUVECs typically observed at wall shear stress of 0.5 dyn/cm^2^, beyond which no tethering was observed; ++, upper limit of tethering observed at wall shear stress of 0.8 dyn/cm^2^; +++, upper limit of tethering observed at wall shear stress of 1.0 dyn/cm^2^; ++++, upper limit of tethering observed at wall shear stress of 2.0 dyn/cm^2^. Data are representative of n > 5 independent experiments.*

Alternatively, the MET state of CSCs (indicated by ALDH expression but not necessarily CD44^+^/CD24^-^ cells; Liu et al., 2012) may regulate E-selectin ligand expression or function. Interestingly, a recent study of all cell lines in **Table 1** except Hs578T revealed that BT-20 and MDA-MB-468 cells, both CD44^+^/CD24^+^ cell lines of the basal A type with relatively high E-selectin ligand activity (**Figure 3** and **Table 1**), possessed the highest percentage of cells with ALDH activity (Deng et al., 2010). CSCs in the MET state may thus maintain or potentially upregulate E-selectin ligands, in contrast to CSCs in EMT. This notion merits further investigation, in that CTCs from breast cancer patients can simultaneously express mesenchymal and stem cell markers in addition to epithelial markers, and not just EMT markers (Aktas et al., 2009; Bonnomet et al., 2010; Armstrong et al., 2011; Kallergi et al., 2011). Moreover, the bone resident chemokine stromal derived factor-1 (SDF-1, **Figure 4**), known to mediate HSC homing and breast cancer migration through ligation of CXCR4, has been shown to regulate miRNAs in breast cancer cells and stromal cells that control breast cancer cell tumorigenicity and quiescence (Lim et al., 2011; Rhodes et al., 2011a,b). It may only be a matter of time until it is shown that SDF-1 also regulates miRNAs associated with EMT-MET phenotypes of CSCs. Thus, bone microenvironment expression of E-selectin, TGF-β, and SDF-1 may beckon a certain type of CTC to establish a metastatic colony: a circulating CSC already equipped to infiltrate the bone parenchyma, or else another cell, CSC or otherwise, that will undergo EMT or MET as needed to attach to endothelium, invade, and grow.

Epithelial-to-mesenchymal transition and MET are, by their very names, dynamic transitional regulators of cellular phenotypes and behaviors. Thus far, we have proposed that these pathways modulate E-selectin ligands on breast CTCs and CSCs. However, it is valid to explore the potential roles of E-selectin and its ligands as regulators of EMT and MET. The proper E-selectin ligands expressed at the right time in the right place (e.g., HCELL, Mac-2bp, and/or glycolipids; Burdick et al., 2006; Shirure et al., 2011, 2012; manuscript in preparation) on a breast CTC in the vasculature at the metastatic site) could facilitate EMT- or MET-generated/maintained CSCs in response to microenvironmental cues. E-selectin-primed cells may then effectively establish metastatic colonies. If a CTC encounters a small capillary rather than a larger venule, another possibility arises. Assuming the CTC can sufficiently deform to enter the capillary, which can be tested *in vitro* in a micropipette assay (**Figure 2**), transit through the capillary with or without E-selectin ligand/E-selectin engagement could also effectively modulate CTC and CSC phenotype. For now, these complex, highly speculative models remain in the theoretical realm, but as separate discoveries are made about CTCs, CSCs, cancer cell mechanical properties, and selectin ligands, comprehensive investigations linking these subjects will become less daunting and perhaps even routine.

Thus, several intriguing theories proposing crosstalk among biochemical and biophysical factors and selectin ligands on CTCs remain to be tested, and are the subject of ongoing collaborative studies in our laboratories. It is anticipated that the results of these investigations will contribute to the fundamental understanding of the cross-regulation of functional selectin ligands with transformative molecular pathways in breast cancer progression, as well as other cancers for which selectins and their ligands are suspected promoters of metastasis.

## BEYOND THE HYPOTHESIZED MODEL

Although this article has been focused on presenting the potential relationships between CSCs, CTCs, and E-selectin ligands in hematogenous distant metastasis, the pathways mediating metastasis in total are far more extensive. Restricting the discussion to the selectins, CTCs may engage P-selectin expressed on blood vascular endothelial cells (Ludwig et al., 2004; Laubli and Borsig, 2010; St Hill, 2012), which is arguably less understood than endothelial E-selectin-mediated pathways. CTCs in the bloodstream may also form multicellular aggregates with platelets and/or leukocytes (Borsig et al., 2002), and the presence of these other cells can alter the manner in which CTCs interact with the vascular endothelium (Kim et al., 1999; Burdick and Konstantopoulos, 2004; Liang and Dong, 2008; Gong et al., 2012). The initial formation of heterotypic aggregates presumably occurs through engagement of P-selectin on platelets or L-selectin on leukocytes with their respective ligands on CTCs (Mannori et al., 1995; Jadhav et al., 2001; McCarty et al., 2002), such as the aforementioned HCELL (Hanley et al., 2005, 2006; Burdick et al., 2006; Barthel et al., 2009), sulfated glycosaminoglycans or proteoglycans (Ma and Geng, 2002; Monzavi-Karbassi et al., 2007; Cooney et al., 2011), or sulfatides (Needham and Schnaar, 1993; Simonis et al., 2010). Perhaps multicellular aggregation induces CSC phenotype(s). This theory warrants further investigation, given the discovery in HB-chips of CTC aggregates indicating prior CTC–leukocyte engagement (Stott et al., 2010), and a recent publication revealing that platelet–cancer cell contact can induce EMT in breast and colon cancer cells (Labelle et al., 2011). Alternatively, completely novel mechanisms of CTC–CSC regulation may be encountered in lymph node metastasis, considering the vastly different biochemical and biophysical environment of the lymphatic system compared to the blood vasculature (Lund and Swartz, 2010; Swartz and Lund, 2012). Thus, other compelling models of CTC–CSC regulation may be proposed and tested, which could lead to new ways to inhibit cancer metastasis.

## CONCLUSION

A full understanding of how a cancer cell progresses from primary tumor cell to CTC to disseminated tumor cell remains elusive. Although EMT, MET, and stem cell pathways are clearly relevant, their effects relative to selectin ligands (and vice versa) on CTCs remain to be determined. On their directed journey to establish new metastatic colonies, CTCs are subject to the influences of a bevy of biochemical and biophysical stressors that may change their phenotype at specific times and at specific locations. CTCs captured from the blood of cancer patients by CellSearch, CTC- or HB-chips, AdnaTest, and other devices reflect only a single temporal data point from which inferences about disease status, treatment strategies, and survival predictions are extrapolated. While this information from blood biopsies is extraordinarily important, some caution is warranted. Molecular markers and phenotypes serving as the basis of capture in these assays have limitations, and information derived from these assays may have further shortcomings in light of CTC dynamism. Therefore, novel CTC capture techniques and therapeutic strategies currently in development must respect the changing epithelial, mesenchymal, CSC-associated, etc., markers and functional phenotypes (e.g., expression of selectin ligands) to be truly meaningful for patients. Ultimately, collective efforts to elucidate the molecular descriptors of CTCs, including selectin ligands and their regulators such as CSC generation/maintenance pathways, will greatly improve the clinical utility of CTCs as diagnostics, prognostics, therapeutic indicators, or therapeutic targets.

## Conflict of Interest Statement

The authors declare that the research was conducted in the absence of any commercial or financial relationships that could be construed as a potential conflict of interest.
